# Orthodontic treatment in impacted maxillary canines. A review of the literature

**DOI:** 10.21142/2523-2754-0904-2021-085

**Published:** 2021-12-09

**Authors:** Alexandra J. Aquino-Valverde, Datfne Milagros Barrientos-Sanchez, Katherine Joselyn Atoche-Socola

**Affiliations:** 1 Carrera de Estomatología, Universidad Científica del Sur. Lima, Perú. aaquinovalverde@gmail.com, datfnebsanz@gmail.com Universidad Científica del Sur Carrera de Estomatología Universidad Científica del Sur Lima Peru aaquinovalverde@gmail.com datfnebsanz@gmail.com; 2 División de Rehabilitación Oral, Carrera de Odontología, Universidad Científica del Sur Lima, Perú. kattyas.22@gmail.com Universidad Científica del Sur División de Rehabilitación Oral Carrera de Odontología Universidad Científica del Sur Lima Peru kattyas.22@gmail.com

**Keywords:** impacted tooth, orthodontic traction. permanent dentition, orthodontic treatment, diente impactado, tracción ortodóntica. dentición permanente, tratamiento de ortodoncia

## Abstract

The prevalence of impacted maxillary canines is 1 to 3%, with approximately 50% of impacted canines causing root resorption of adjacent teeth. If the permanent canine has not acquired its correct position, evaluation by an orthodontist is necessary to determine the most adequate treatment. Surgery and orthodontic treatment are used for the treatment of impacted canines in the maxilla affecting permanent dentition. Selection of the most adequate treatment to achieve the correct position of the canine tooth depends on the position of the canine. This article summarizes the different techniques available and describes their advantages, and cost-benefit considerations.

The objective of this literature review is to describe the etiology of impacted maxillary canines and provide an update of the information on orthodontic treatments available for these patients.

## RESUMEN

La prevalencia de caninos maxilares impactados es del 1 al 3%. Aproximadamente, el 50% de los caninos impactados provocan la reabsorción radicular de los dientes adyacentes. Por otro lado, si el canino permanente no ha comenzado a encontrar su posición correcta, se debe considerar como primera opción la evaluación de un ortodoncista, quien brindará diversos tratamientos. La cirugía y el tratamiento de ortodoncia se utilizan para tratar los caninos impactados en el maxilar afectado en la dentición permanente. Según la posición del canino, se puede seleccionar el mejor tratamiento para lograr la ubicación correcta del canino. Este artículo resume todas estas técnicas diferenciales, conociendo sus ventajas y consideraciones de costo-beneficio.

El objetivo de esta revisión de literatura es actualizar la información sobre tratamientos de ortodoncia en caninos maxilares impactados, describir la etiología de caninos maxilares impactados, así como describir la tracción y los tratamientos de ortodoncia para caninos maxilares impactados.

## INTRODUCTION

Orthodontists frequently present many complications when treating impacted maxillary canines; this condition of impaction has a prevalence of approximately 1% to 3%.^1^^-^^3^ Furthermore, canine impaction is 2- to 3-fold more prevalent in women compared to men. ^2^ In the absence of timely treatment, the presence of impacted maxillary canines can lead to problems in the oral cavity, such as root resorption of the surrounding incisors or those close to the eruption site. Therefore, early, adequate treatment with traction of the impacted canine by an orthodontist is essential in order to avoid possible adverse effects. ^3^

The cause of maxillary canine impaction remains under debate. Some studies have described genetic inheritance as the most likely cause, while others have suggested the eruption guide theory which is related to a lateral incisor manifesting a small root or the absence of the lateral incisor. ^4^^,^^5^

Impaction of the maxillary canine can be determined by different approaches, the most accepted being that proposed by Erikson and Kurol, in which the measurement of the impact angle of the longitudinal axis of the canine is evaluated with respect to the center line of the tooth, or by panoramic radiographic images. Regarding the premolars, lateral incisors and central incisors, this axis would be located in the shaded areas at the tips of the canines, obtaining impaction areas ranging from sectors 1 to 5, with sector 1 being close to the premolars. When sector 5 is close to the midline of the tooth, the collision height can also be measured, thereby indicating the distance from the tip of the canine tooth to the occlusal plane. ^6^

It is important to determine the etiology of canine impaction in the maxilla in order for orthodontists to achieve a correct diagnosis and provide effective treatment, and updated information on the current diagnostic and treatment approaches is key to achieve this goal. Therefore, this literature review aims to describe the etiology and the treatments currently used to effectively treat impacted canines in the maxilla.

## MATERIAL AND METHODS

Extensive research has been done in the existing literature on this topic. From the beginning of this information in Medline until October 9, 2020, the bibliographic search was carried out including articles published in the Medline database through PubMed, SCOPUS, SCIENCE DIRECT, WILEY and SCIELO with no language restriction. The keywords used were: impact, maxillary canine, orthodontic treatment, MIC and retained canine. We included observational, descriptive, longitudinal research, systematic reviews, books, and editorials. 

## RESULTS AND DISCUSSION

Thirty-nine articles were included in this review.

## ETIOLOGY OF IMPACTED MAXILLARY CANINES

The most common causes of impacted canines include endocrine disorders, such as growth hormone or thyroid hormone deficiency, and vitamin A and D deficiency, as well as congenital syphilis, temporomandibular joint ankylosis and genetic inheritance. Local causes involve the lack of space, dislocation of adjacent teeth, early detachment of deciduous teeth, abnormal curvature of the root caused by trauma in childhood, and the development of diseases of the tooth germ and periodontitis. ^7^^-^^11^ Lappin described that canines were affected by blockage due to an excess of tooth or dentinoma, recent trauma, or eruptive changes in the canines on the same side. ^12^ In the absence of controlled studies to establish the cause of this anomaly it has been suggested that it is due to the lack of reabsorption of the temporal canine, and several studies have reported that timely removal of temporary canines seems to be very useful for inducing adequate eruption of permanent canines. ^12^^-^^15^ A recent study showed that impaction of the lateral incisor can lead to obstruction of supernumerary teeth, odontomas or lesions, with a subsequent high frequency of eruptive alteration of the canine on the same side.^16^ This study showed a significant increase in the prevalence and severity of displaced canines of 41.3%, with buccal displacement being observed in 30.2%, palatal displacement in 9.5%, and canine-lateral transposition of incisors in 1.6% of patients. ^17^ Several studies have associated canine impaction in the maxilla with the absence of permanent lateral incisors, reporting that the presence of these teeth, in this case the atypical lateral incisors, caused deviation of the maxillary canines around their correct position. ^18^ Displacement of the maxillary canines has two possible processes. The first is developmental related to the lack of orientation of the lateral incisors, leading to the development of a new path for the descending trajectory of the anterior side, and the second is related to the late stage of the tooth entering the narrow part of the alveolar process. ^19^ The treatment of choice to correct maxillary canines in palatal eruption in individuals aged 10 to 13 years is extraction of the temporal canine provided that tooth spacing is correct, since 91% of canine teeth, overlap the adjacent lateral incisor root, while 64% of canine teeth overlap the lateral incisor relative to the middle of the root. ^20^

## ORTHODONTIC TRACTION AND TREATMENTS FOR IMPACTED MAXILLARY CANINES

Traction is more appropriate in cases diagnosed early, such as in infants since they are in their growth stage. Orthodontic traction consists of surgically exposing the affected tooth, and then performing traction to guide the tooth and align it with the dental arch. Loss of bone around tooth extraction, root resorption, and recession of the gums are the most common complications of this type of surgery. ^21^ Surgical tooth displacement requires discussion between the orthodontist and the physician to determine the most adequate approach to carry out, considering the depth of action, the anatomy of the edentulous area and the type of orthodontic force to be used. ^22^ When the tooth is impacted in the middle third of the socket, closed treatment is strongly recommended. In this case, it is necessary to remove as little bone tissue as possible by exposing the crown of the tooth to hold the traction accessory. To avoid placing the traction in a very high gingival position, lateral traction is usually necessary, and special care must be taken in relation to the direction of traction. ^23^ The most common traction method for canines involves surgical exposure and placement of orthodontic accessories to apply slow, light force to move the teeth into the correct position. ^24^ Another treatment alternative is the use of heavy anchorage with a transpalatal arch to give reinforcement in a variety of applications ranging from orthodontic applications as well as in common dental treatments. A stainless steel transpalatal arch can be used as an anchoring device that is attached to molars and premolars to orthodontically position the teeth through the palate. ^25^

## TREATMENT WITH TIE WIRES IN CROSSBOW

This is composed of round wires that accumulate force when bent on the longitudinal axis and have a horizontal cross section that enters the double tube placed in the brackets system. The arc force is relative to the length of the wire in the vertical and horizontal directions. Once the affected tooth is clinically and radiologically identified, surgery is performed to expose part of the enamel surface of the affected tooth. Contact with soft tissue contact is made to expose the bone covering the crown. The roots of the adjacent teeth should not be touched, since hardening of the cement will affect root development.^26^ Canine teeth are generally fixed or in a variable position, and therefore, an orthodontic button with self-curing resin can be placed, although protection of the adhered gums produced by the impact of buccal dentin fragments must be taken into account. If the tooth is affected by bone, fibrous adhesions around the tooth must be avoided. ^27^ This treatment will control the germination of the teeth by applying a certain force in the vestibular direction, so that the crown of the canines can move away from the root, thus avoiding resorption. This will also allow exerting controlled and easily modifiable forces. This system can overcome the limitations of mobile systems, such as traction of the upper jaw hitting the teeth. Moreover, it will reduce the root resorption of the adjacent teeth because it moves the teeth towards the buccal side for later repositioning.^28^

## MICROINPLANT TREATMENTS

The advantages of microscrews over osseous integration implants have led to their increasing popularity as a source of bone anchorage in modern orthodontics.^29^ In many clinical situations, the strength and stability of microscrew implants are sufficient to move teeth without losing mutual anchorage. The article by Park et al. describes the potential of microimplant anchoring in the germination of canine orthodontic teeth.^30^

X-rays taken at different angles should be used to visualize the location of the canine. This technique is not intended to produce root movement, and therefore, it is not suitable for canine teeth that impact horizontally or canine teeth that have a straighter root at the crown. ^31^ Due to the lack of alveolar bone in the canine area, small implants should be used, especially after the original canine tooth has been extracted. Since the force required to tighten the tooth is less than 50g, a miniscrew is ideal. The crown and root of the tooth can be drawn on the processed mold to determine the direction of force required to insert the tooth into the dental arch. ^32^ Microimplants should be placed in the alveolar bone of the labial cortex with this line of force, at an angle of 10-20° with respect to the bone surface and as parallel as possible to the longitudinal axis of the tooth. This procedure maintains the tip of the microscrew on the buccal side and reduces the possibility of the microscrew coming into contact with the root of the tooth. ^33^ Although the head of the microimplant should be placed as tangentially as possible to maximize the vertical component of force, implants placed too high can become unstable due to the accompanying increased torque. The attachment is attached to the labial surface of the affected canine to allow the teeth to rotate without excessive rotation. After the canine enters the arch, tongue support can be added for more precise control. ^34^

## TREATMENTS WITH REINFORCED ANCHORAGE

Reinforced anchorage is another method of treatment of maxillary canines, the objective of which is to prevent secondary effects after orthodontic traction. This treatment requires a heavy buccal arch with a bracket slot and palatal anchorage in the maxillary arch. ^35^^)^ Therefore, to obtain traction of the affected maxillary canines, it is often necessary to use heavy anchors and tensioned arch wires in the maxillary arch to preclude the adverse effects of traction. Also, to resolve the effects of the canines, adequate force is required to move them through the bones. ^36^ After orthodontic traction with reinforced anchors, the use of copper titanium wire arches for the alignment and correction of the segments in the areas of incisors, premolars and molars, metal supports are used for the durability of the deciduous canine (if present). This space is equipped with an open helical spring between the lateral incisor and the nickel-titanium arch wire, being essential requirements before surgery. ^37^ A temporary rigid anchor is then installed over the bands of the first permanent molars accompanied by a rigid palatal acrylic button and arch wire on all the palatal surfaces of all maxillary teeth, or a stainless-steel wire with multiple palatal and occlusal buccal hooks can also be placed. This anchor is cemented at least 4 weeks before surgery. Buccal hooks and anchor extensions allow the buckles of the closed coil nickel-titanium springs to be attached, and trans alveolar intraosseous traction is performed, thus preventing the springs from immersing themselves in the attached gingiva and mucoperiosteum, limiting activation. The stainless-steel dental arch in the bracket placed on the tooth is aligned and level, making dental arch suitable for the final molar to be anchored. ^38^ The buccal side of each fixed canine is secured with a closed coil nickel-titanium spring and activated every 4 to 8 weeks as required by orthodontists. After traction of the canine is achieved, the bone anchor is removed after the incisors and premolars have been stabilized and protected. Thereafter, the steps of intercuspation and completion of the orthodontic treatment is carried out completing the orthodontic treatment. ^39^

## CONCLUSION

Surgery and orthodontic treatment are used to treat impacted canines in the maxilla affecting permanent dentition. Before starting orthodontic treatment, it is advisable to first extract the canines from the root of the incisor and then bring the dental arch inwards. The most adequate treatment to achieve correct positioning of the canine tooth is selected according to the position of the canine. Treatments of the impacted canine have a very limited effect on periodontal disease.

## References

[1] Alqerban A (2019). Impacted maxillary canine in unilateral cleft lip and palate A literature review. Saudi Dent J.

[2] Oz AZ, Ciger S (2018). Health of periodontal tissues and resorption status after orthodontic treatment of impacted maxillary canines. Niger J Clin Pract.

[3] Arriola-Guillén LE, Aliaga-Del Castillo A, Ruiz-Mora GA, Rodríguez-Cárdenas YA, Dias-Da Silveira HL (2019). Influence of maxillary canine impaction characteristics and factors associated with orthodontic treatment on the duration of active orthodontic traction. Am J Orthod Dentofacial Orthop.

[4] Cruz RM (2019). Orthodontic traction of impacted canines Concepts and clinical application. Dental Press J Orthod.

[5] Grybiene V, Juozenaite D, Kubiliute K (2019). Diagnostic methods and treatment strategies of impacted maxillary canines A literatura review. Stomatologija.

[6] Ericson S, Kurol J (1986). Radiographic assessment of maxillary canine eruption in children with clinical signs of eruption disturbance. Eur J Orthod.

[7] Pignoly M, Monnet-Corti V, Le Gall M (2016). Échec de la mise en place de dents retenues et incluses (Reason for failure in the treatment of impacted and retained teeth). Orthod Fr.

[8] Iancu Potrubacz M, Chimenti C, Marchione L, Tepedino M (2018). Retrospective evaluation of treatment time and efficiency of a predictable cantilever system for orthodontic extrusion of impacted maxillary canines. Am J Orthod Dentofacial Orthop.

[9] Alejos-Montante K, Martínez-Zumarán A, Torre-Delgadillo G, Rosales-Berber MÁ, Garrocho-Rangel A, Pozos-Guillén A (2019). Early identification of permanent maxillary canine impaction A radiographic comparative study in a Mexican population. J Clin Exp Dent.

[10] Brusveen EM, Brudvik P, Boe OE, Mavragani M (2012). Apical root resorption of incisors after orthodontic treatment of impacted maxillary canines a radiographic study. Am J Orthod Dentofacial Orthop.

[11] Elhaddaoui R, Benyahia H, Azeroual MF, Zaoui F, Razine R, Bahije L (2016). Resorption of maxillary incisors after orthodontic treatment--clinical study of risk factors. Int Orthod.

[12] Lappin MM (1951). Practical management of the impacted maxillary canine. Am J Orthod.

[13] Schroeder MA, Schroeder DK, Capelli J, Santos DJDS (2019). Orthodontic traction of impacted maxillary canines using segmented arch mechanics. Dental Press J Orthod.

[14] Dagsuyu IM, Kahraman F, Oksayan R (2018). Three-dimensional evaluation of angular, linear, and resorption features of maxillary impacted canines on cone-beam computed tomography. Oral Radiol.

[15] Shapira Y, Kuftinec MM (1998). Early diagnosis and interception of potential maxillary canine impaction. J Am Dent Assoc.

[16] Izadikhah I, Cao D, Zhao Z, Yan B (2020). Different management approaches in impacted maxillary canines an overview on current trends and literature. J Contemp Dent Pract.

[17] Becker A, Smith P, Behar R (1981). The incidence of anomalous maxillary lateral incisors in relation to palatally-displaced cuspids. Angle Orthod.

[18] Jacobs SG (1994). Palatally impacted canines aetiology of impaction and the scope for interception. Report of cases outside the guidelines for interception. Aust Dent J.

[19] Becker A, Chaushu S (2015). Etiology of maxillary canine impaction a review. Am J Orthod Dentofacial Orthop.

[20] Ericson S, Kurol J (1988). Early treatment of palatally erupting maxillary canines by extraction of the primary canines. Eur J Orthod.

[21] Manne R, Gandikota C, Juvvadi SR, Rama HR, Anche S (2012). Impacted canines Etiology, diagnosis, and orthodontic management. J Pharm Bioallied Sci.

[22] Kokich VG (2004). Surgical and orthodontic management of impacted maxillary canines. Am J Orthod Dentofacial Orthop.

[23] Pavlidis D, Daratsianos N, Jager A (2011). Treatment of an impacted dilacerated maxillary central incisor. Am J Orthod Dentofacial Orthop.

[24] Bedoya MM, Park JH (2009). A review of the diagnosis and management of impacted maxillary canines. J Am Dent Assoc.

[25] Pizzoni L (2010). Fibre reinforced composite transpalatal arch in impacted canine orthodontic treatment. Prog Orthod.

[26] Andreasen JO, Andreasen FM (2000). Essentials of traumatic injuries to the teeth..

[27] Oberoi Snehlata, Knueppel Stephanie (2012). Three-dimensional assessment of impacted canines and root resorption using cone beam computed tomography. Oral Surgery, Oral Medicine, Oral Pathology and Oral Radiology.

[28] Jacoby H (1979). The "ballista spring" system for impacted teeth. Am J Orthod.

[29] Costa A, Raffainl M, Melsen B (1998). Miniscrews as orthodontic anchorage a preliminary report. Int J Adult Orthodon Orthognath Surg.

[30] Park HS, Kyung HM, Sung JH (2002). A simple method of molar uprighting with micro-implant anchorage. J Clin Orthod.

[31] Gutiérrez Labaye P, Hernández Villena R, Perea García MA, Escudero Castaño N, Bascones Martínez A (2014). Microtornillos una revisión. Avances en Periodontología.

[32] Gray JB, Steen ME, King GJ, Clark AE (1983). Studies on the efficacy of implants as orthodontic anchorage. Am. J. Orthod.

[33] Park HS (1999). The skeletal cortical anchorage using titanium microcrew implants. Kor. J. Orthod.

[34] Kanomi R (1997). Mini-implant for orthodontic anchorage. J. Clin. Orthod.

[35] Arriola-Guillén LE, Ruiz-Mora GA, Rodríguez-Cárdenas YA, Aliaga-Del Castillo A, Dias-Da Silveira HL (2018). Root resorption of maxillary incisors after traction of unilateral vs bilateral impacted canines with reinforced anchorage. Am J Orthod Dentofacial Orthop.

[36] Heravi F, Shafaee H, Forouzanfar A, Zarch SH, Merati M (2016). The effect of canine disimpaction performed with temporary anchorage devices (TADs) before comprehensive orthodontic treatment to avoid root resorption of adjacent teeth. Dental Press J Orthod.

[37] Yadav S, Chen J, Upadhyay M, Jiang F, Roberts WE (2011). Comparison of the force systems of 3 appliances on palatally impacted canines. Am J Orthod Dentofacial Orthop.

[38] Killiany DM (1999). Root resorption caused by orthodontic treatment an evidence-based review of literature. Semin Orthod.

[39] Schmidt AD, Kokich VG (2007). Periodontal response to early uncovering, autonomous eruption, and orthodontic alignment of palatally impacted maxillary canines. Am J Orthod Dentofacial Orthop.

